# Severe maternal morbidity: a case-control study in Maranhao, Brazil

**DOI:** 10.1186/1742-4755-10-11

**Published:** 2013-02-11

**Authors:** Ana Paula Pierre de Moraes, Sandhi Maria Barreto, Valeria Maria A Passos, Patricia S Golino, Janne E Costa, Marina X Vasconcelos

**Affiliations:** 1 Doctor of Public Health / Intensive care physician, Universidade Federal do Maranhao, Rua Barao Itapary, 32 (UTI geral)-Centro, Sao Luis, Maranhao cep 65020-070, Brazil; 2 Centro de Pos-graduacao Belo Horizonte, Universidade Federal de Minas Gerais (UFMG), Av Professor Alfredo Balena 190 – 5 andar, Belo Horizonte, Minas Gerais, cep 30130-100, Brazil; 3 Ceuma Universidade - Unidade Renascença, Rua Josue Montello, n 1, Renascença II, Sao Luis, Maranhao, CEP 65.075-120, Brazil; 4 University Hospital, Universidade Federal do Maranhao (UFMA), Unidade Materno Infantil, Rua Silva Jardim, n 215, Centro - Sao Luis-MA, Sao Luis, Maranhao, CEP: 65020-560, Brazil

**Keywords:** Complicações da gravidez, Mortalidade materna, Puerpério, Prenatal, Gravidez, Estudo caso-controle, Pregnancy complications, Maternal mortality, Puerperium, Prenatal care, Pregnancy, Case–control study

## Abstract

**Background:**

Maternal mortality and morbidity are among the top public health priorities in Brazil, being quite high, especially among the most disadvantage women. A case control study was developed to identify risk factors for severe maternal morbidity in Sao Luis, one of the poorest Brazilian State Capitals.

**Methods:**

The case–control study was carried out between 01/03/2009 and 28/02/2010 in two public high-risk maternities facilities and in two intensive care units (ICUs) for referral of obstetric cases. All cases hospitalized due to complications during gestation period, childbirth or up to 42 days of puerperium and who fulfilled any of Mantel's and/or Waterstone's criteria were identified. Two controls per case were randomly selected among patients of the same clinics discharged for other reasons. Data were obtained through a structured interview as well as from medical charts and prenatal cards and included sociodemographic variables, clinical and obstetric histories, behavioral factors and exposure to stress factors during pregnancy, pre-natal assistance and obstetric complication and childbirth care.

**Results:**

In the final model of the unconditional logistic regression analysis, being older than 35 years (OR=3.11; 95% CI:1.53-6.31), previous hypertension (OR=2.52; 95% CI:1.09-5.80), history of abortion (OR=1.61; 95% CI:0.97-2.68), 4–5 pre-natal consultations (OR=1.78; 95% CI:1.05-3.01) and 1–3 pre-natal consultations (OR=1.89; 95% CI:1.03-3.49) were independently associated with severe maternal morbidity.

**Conclusions:**

The results corroborate the importance of reproductive healthcare, of identifying a high-risk pregnancy and of a qualified and complete prenatal care to prevent severe morbid events.

**Resumo:**

**Introdução:**

A mortalidade e morbidade maternas estão entre os tópicos prioritários da Saúde Pública brasileira, especialmente na população de menor nível socioeconômico. Um estudo caso-controle foi desenvolvido para identificar os fatores de risco para morbidade materna grave em São Luís, capital de um dos estados mais pobres do Brasil.

**Método:**

Estudo caso-controle realizado em duas maternidades públicas de alto risco e duas UTIs de referência aos casos obstétricos entre 01/03/2009 e 28/02/2010. Foram incluídas todas as pacientes internadas por complicação do período grávido-puerperal e que preenchiam os critérios de Waterstone e/ou Mantel para morbidade materna grave. Foram selecionados para cada caso, dois controles por sorteio aleatório dentre as pacientes internadas no mesmo período e mesma maternidade que o caso. As informações de domínio sociodemográfico, clínico, obstétrico, comportamental, exposição a eventos estressores na gestação, assistência ao pré-natal, intercorrências obstétricas e atenção ao parto, foram obtidas por meio de entrevista estruturada. As variáveis foram analisadas por modelo de regressão logística múltipla não condicional, baseado em modelo hierarquizado a *priori*.

**Resultados:**

Foram identificados como fatores de risco para morbidade materna grave: idade >35 anos (OR=3,11; IC 95%:1,53-6,31), hipertensão prévia à gestação (OR=2,52; IC 95%:1,09-5,80), antecedente de aborto (OR=1,61; IC 95%:0,97-2,68), ter realizado 4–5 consultas pré-natais (OR=1,78; IC 95%:1,05-3,01) ou 1–3 consultas (OR=1,89; IC 95%:1,03-3,49).

**Conclusão:**

Os resultados do estudo corroboram a importância da assistência à saúde reprodutiva e o pré-natal completo e qualificado na prevenção de eventos mórbidos graves durante o ciclo grávido-puerperal.

## Background

The reduction of maternal mortality is one of the United Nations’ Millennium Development Goals [1]. Maternal death has serious social repercussions, which affect individuals and families. Maternal mortality is usually associated with deficiencies in the health care system and can be, therefore, avoided. For this reason, maternal mortality is considered to be a powerful socio-economic and health indicator within a population, being inversely related to the level of human development. In spite of this consideration and notwithstanding the variety of maternal mortality ratios throughout the world, maternal death is a rare event and the isolated study of this phenomenon is not capable of reflecting the quality of obstetrical assistance. In order to improve this situation, there are new indicators based on studies on severe maternal morbidity, which is an important complementary indicator for permanent surveillance of maternal health [2].

Severe maternal morbidity or *near miss* refers to any severe complication during gestation, childbirth or puerperium [3-6]. Pregnant women who endured a *near miss* situation have a profile probably similar to those who progress towards death, representing, thus, a proxy model for maternal mortality [7,8]. To clarify how the morbid conditions could have lead to death creates the opportunity to improve the assistance to women at risk [9].

In Brazil, maternal mortality is still elevated (77.2/100,000 live births in 2006) [10]. In Sao Luis, the State Capital with the lowest income per capita in the country [11], maternal mortality is even higher than the estimated for the country (84.6/100,000 live births in 2006) [12]. We conducted a previous cohort study which showed that obstetric reasons were the main causes of severe maternal morbidity in the city [13]. The objective of the present study is to identify the risk factors for severe maternal morbidity in the same population.

## Methods

A case–control study was carried out to identify risk factors for severe maternal morbidity in public maternities in Sao Luis, Capital of Maranhão, with a population estimated at one million people, 85% of whom depend exclusively on the health care provided by the public national system [14].

Data were collected between March 1, 2009, and February 28, 2010. In order to seize the greatest number of severe maternity morbidity cases, we assessed all inpatients at the two public maternity clinics for high risk patients and the two general ICUs which are references of obstetric cases. There are no obstetric ICUs in the city. The two selected maternities are responsible for approximately 8,000 births per year, corresponding to half of the total number of deliveries performed in all seven existing public clinics.

All patients admitted with complications during gestation period, childbirth or up to 42 days of puerperium and who fulfilled at least one of the requirements proposed by Mantel’s [5] and/or Waterstone’s [15] criteria were considered cases of severe maternal morbidity (Table 1). The World Healtlh Organization criteria for maternal near miss [16] were not evaluated in this work because they were published after the beginning of the data gathering. Four trained doctors identified the cases by searching medical charts and consulting directly the health professionals who worked in these institutions at least three times a week during the entire period of the study.

**Table 1:** Criteria for severe maternal morbidity

**MANTEL **[5]	**WATERSTONE **[15]
Acute pulmonary oedema: Necessitating intravenous furosemide or intubation	Severe pre-eclampsia
Cardiac arrest	Eclampsia
Hypovolaemia requiring ≥ 5 unit of packet red blood cells	HELLP Syndrome ((haemolysis, elevated liver enzymes, low platelets)
Intensive care admission for sepsis	Severe sepsis
Emergency hysterectomy for sepsis	Severe haemorrhage (estimated blood loss ≥ 1500 ml, peripartum fall in a hemoglobin concentration ≥ 4g/dl or transfusion ≥ 4 units of packed red blood cells)
Intubation and ventilation for more than 60 minutes, for ant reason other than a general anesthetic	Uterine rupture
Peripheral O2 saturation <90% for more than 60 minutes	
Ratio (PaO2/FiO2) <300	
Oliguria, defined as diuress <400ml/24h refractory to careful hydration or to furosemide or dopamine	
Acute urea deterioration to > 15 mmol/ l or creatinine > 400mmol / l	
Jaundice in the presence of pre-eclampsia	
Diabetic ketoacidosis	
Thyrotoxic crisis	
Acute thrombocytopenia requiring a platelet transfusion	
Coma in a patient lasting > 12 hours	
Subarachnoid or intracerebral hemorrhage	
Intensive care admission admission for any reason	
Emergency hysterectomy for any reason	
Anaesthetic Accident: severe hypotension (systolic pressure <90 mmHg lasting >60 minutes) associated with spinal or epidural anaesthetic	
Anaesthetic Accident : Failed tracheal intubation requiring anaesthetic reversal	

Using simple random sampling, two controls were selected for each case. All the patients who did not fulfil the criteria for severe maternal morbidity and who were hospitalized in the same maternity clinic where the cases occurred or had been discharged during the same period were eligible as controls. Patients who did not reside in the city were excluded.

The sample size calculated as 115 cases and 230 controls allowed estimating an Odds Ratio equal or greater than 2.5, with 90% power and 95% confidence interval, considering a 16% prevalence of exposure (less than four prenatal visits) among controls. The expected prevalence of inadequate prenatal care – the main variable of interest in this study – was estimated using the Ministry of Health data system [17].

The risk factors were classified as 1) sociodemographic, 2) clinical and obstetric history, 3) behavioural and stress events during the current pregnancy, 4) prenatal care, 5) current obstetric complications and 6) childbirth assistance. The data was obtained through a structured interview applied after obtaining a signed free and informed consent and before hospital discharge. The sociodemographic and health assistance data was also obtained from medical charts and prenatal cards.

**Figure 1:**
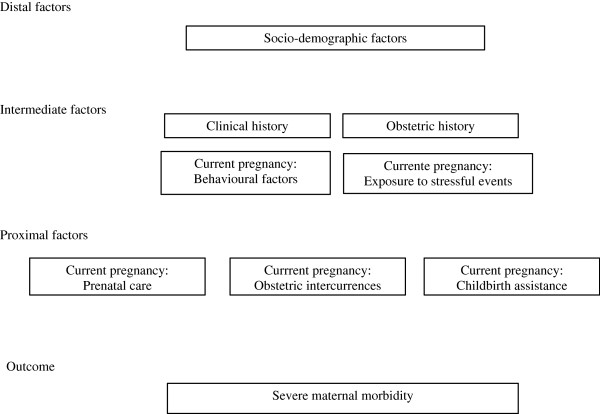
Hierarchical model designed for the multivariate analysis of risk factors for severe maternal morbidity.

Data were stored using Epi-Data program, version 3.1. The unconditional multivariate logistic analysis was carried out following a hierarchical order of variables defined *a priori* (Figure 1). The object of this analysis method was to test the hypothesis using concepts of proximal and distal determinants of the examined phenomenon [18,19]. We considered the hierarchical level of each group of variables in the chain of social determinants of severe maternal morbidity, where the more distal factors have a greater influence than the more proximal ones.

In the multivariable analysis, all variables in each group statistically associated with the outcome at the level of p<0.20 were considered following the hierarchical structure indicated above. From the second group of variables on, after adjusting for the previous groups, the variables associated at the level of p<0.10 were kept in the model. After the inclusion of the last group of variables, only those factors which remained statistically significant (p-value<0.05) were kept. The magnitude of the association between the exposure variables and the outcome was estimated by the *odds ratio* and its respective 95% confidence intervals. The analysis was performed using the statistic software Stata (version10).

The study was approved by the Research Ethics Committee of the Federal University of Minas Gerais (ETIC no: 589/08).

Results

We identified 127 cases of severe maternal morbidity during the study period. Five cases (3.9%) of severe maternal morbidity were lost, three of whom refused to participate. Therefore, 122 women diagnosed with severe maternal morbidity participated in the study: 66.4% severe pre-eclampsia; 11.5% eclampsia; 11.4% obstetric haemorrhage; 5.7% HELLP syndrome; 2.5% infected abortion, 1.6% pre-eclampsia superimposed upon chronic hypertension; and 1.6% non-obstetric complications. Three of the 244 selected controls were substituted by random sampling; two of them declined to participate and one was excluded for presenting a clinical picture similar to seizure.

In the study period, the maternal mortality in the city was 84.5/100,000 live births [12]. Table 2 shows the distribution of cases and controls according to selected sociodemographic and clinical variables, distal and intermediate factors in the hierarchical model, respectively. Age equal or greater than 35 years, being at the lower tertile of the distribution of per-capita monthly income, and history of hypertension and abortion were statistically associated with being a case. Cases and controls did not differ with regard to schooling, working, marital status, home environment, presence of comorbidities, number of gestations, tobacco smoking, physical activity and exposure to stressful life events during the pregnancy. About 80% of the cases and controls did not plan the current pregnancy, but there was no statistical difference between them.

**Table 2:** **Distribution of controls and cases and controls of severe maternal morbidity according to selected socio**-**demographic and clinical factors**

**Distal and intermediate variables**	**Cases**	**Controls**	**χ2**	**OR (95% IC)**	***p****-****value***
	**n (%)**	**n (%)**			
**Age**					
20-34	80 (65.6)	174 (71.3)		1.00	
<20	18 (14.8)	54 (22.1)		0.73 (0.40-1.32)	
≥35	24 (19.7)	16 (6.6)		3.26 (1.64-6.48)	
			**15**.**43**		<**0**.**001**
**Per capita monthly income**					
Upper tertile	30 (24.6)	79 (32.4)		1.00	
Middle tertile	39 (3.0)	88 (36.1)		1.17 (0.66-2.05)	
Lower tertile	49 (40.2)	69 (28.3)		1.87 (1.07-3.26)	
Not informed*	4 (3.3)	8 (3.3)			
			**5**.**61**		**0**.**06**
**Previous hypertension**					
No	106 (86.9)	232 (95.1)		1,00	
Yes	16 (13.1)	12 (4.9)		2.91 (1.33-6.38)	
			**7**.**74**		<**0**.**01**
**Age at the first pregnancy (years)**					
≥20	66 (54.1)	111 (45.5)		1.00	
16-19	45 (36.9)	94 (38.5)		0.80 (0.50-1.28)	
<16	11 (9.0)	39 (16.0)		0.47 (0.22-0.98)	
			**4**.**39**		**0**.**11**
**Previous abortion**					
No	80 (65.6)	187 (76.6)		1.00	
Yes	42 (34.4)	57 (23.4)		1.72 (1.06-2.77)	
			**5**.**04**		**0**.**03**
**Physical activity during gestation period (leisure time)**					
No activity	93 (76.2)	160 (65.6)		1.00	
1-2 x week	15 (12.3)	44 (18.0)		0.58 (0.31-1.11)	
≥ 3 x week	14 (11.5)	40 (16.4)		0.60 (0.31-1.16)	
			**4**.**33**		**0**.**11**

* The missing information was not considered for the assessment of **χ2**.

São Luis, Brazil, 2009.

With regard to the obstetric variables, the proximal factors, the great majority (94%) of the participating women reported prenatal care. Cases had a higher risk of having had less than six consultations during the gestation period, but the statistical significance was borderline at the level of p<0.10. Previous hypertension was five times more frequent among cases than controls. Cases and controls did not differ regarding to history of gestational haemorrhage at any period or excessive weight gain. The chance of being referred to a high risk care was four times greater among cases than controls (Table 3).

**Table 3:** Distribution of controls and cases of maternal morbidity according to selected obstetric factors

**Proximal variables**	**Cases n (%)**	**Controls n (%)**	**χ2**	**OR (95% CI)**	***p****-****value***
**Number of consultations**					
≥ 6	51 (41.8)	135 (55.3)		1.00	
4-5	42 (34.4)	67 (27.5)		1.65 (1.00-2.74)	
1-3	19 (15.6)	27 (11.1)		1.86 (0.95-3.63)	
0	9 (7.4)	13 (5.3)			
Not informed*	1 (0.8)	2 (0.8)			
			**6**.**14**		**0**.**11**
**Hypertension during gestation**					
No	72 (59.0)	207 (84.8)		1.00	
Yes	41 (33.6)	24 (9.8)		4.84 (2.73-8.50)	
No prenatal care /not informed*	9 (7.4)	13 (5.3)			
			**33**.**20**		<**0**.**001**
**Reference to high risk prenatal care**					
No	71 (58.2)	202 (82.8)		1.00	
Yes	42 (34.4)	29 (11.9)		4.12 (2.39-7.11)	
No prenatal care/ not informed*	9 (7.4)	13 (5.3)			
			**28**.**07**		<**0**.**001**

* The missing information was not considered for the assessment of **χ2.**

Sao Luis, Brazil, 2009.

Table 4 presents the final model after the unconditional hierarchical multiple logistic regression analysis. The factors “hypertension during pregnancy” and “reference to high risk pregnancy care” were not included in multivariable analysis due to their extreme proximity with the outcome and, consequently, to the risk of being confused with case definition itself. The following factors remained statistically associated at the end of the analysis: age equal or over 35 years (OR=3.11; 95% CI:1.53-6.31), previous hypertension (OR=2.52; 95% CI:1.09-5.80), history of abortion (borderline significance–OR=1.61; 95% CI:0.97-2.68), 4–5 prenatal consultations (OR=1.78; 95% CI:1.05-3.01) and 1–3 prenatal consultations (OR=1.89; 95% CI:1.03-3.49).

**Table 4:** Risk factors for severe maternal morbidity in the multivariable analysis

**Multivariate analysis according to the hierarchical model**	**OR**	**Adjusted OR**	**95% CI**	***p****-****value***
**Age**				
20-34	1.00	1.00		
< 20	0.73	0.73	0.39-1.37	
≥ 35	3.26	3.11	1.53-6.31	< 0.01
**Previous hypertension**				
No	1.00	1.00		
Yes	2.91	2.52	1.09-5.80	0.03
**History of abortion**				
No	1.00	1.00		
Yes	1.72	1.61	0.97-2.68	0.07
**Number of consultations**				
≥ 6	1.00	1.00		
4-5	1.65	1.78	1.05-3.01	0.03
1-3	1.86	1.89	1.03-3.49	0.04

Sao Luis, 2009.

## Discussion

In general, cases and controls were young, from very low income families, married and had at least 8 years of schooling. Among the great number of variables investigated, we identified four risk factors for severe maternal morbidity: age equal or over 35 years, previous history of hypertension, history of abortion and having had less than six prenatal consultations, minimum number of prenatal visits recommended by the Brazilian Ministry of Health.

Higher risk of severe maternal morbidity for older women had already been identified in prior studies [8,15] Age is not amenable to change, but it is useful to identify women who require extra vigilance of maternal and fetal risks.

Lower monthly income per-capita was not associated with being a case in the final model, after considering the effect of prenatal and hospital assistance. Waterstone and cols (2001) [15], on the other hand, showed an independent association between social exclusion – characterized, among others, by age under 16 years, low income and poor living condition – and severe maternal morbidity. However, the participants of this study are all very poor and such homogeneity regarding socioeconomic aspects has probably prevented finding differences between cases and controls.

A previous history of hypertension has also been reported as a risk factor for severe maternal morbidity [15,20]. In Brazil, a study performed in an obstetric ICU reported that 19% of the patients had some pre-existing clinical condition, including chronic hypertension [21]. Chronic hypertension is one of general medical situations which should be consider to refer a pregnant woman to a high risk pregnancy care, as they require permanent vigilance [22].

About one third of cases and one fourth of controls reported having had a previous abortion. These prevalences were much higher than the rate reported for the whole country (16.2%) [23] .A research carried out in the Brazilian capitals, identified abortion as the third cause of maternal death [24]. In another Brazilian study, the history of abortion was associated with the occurrence of severity of maternal morbidity for the characterization of near miss [25]. The history of abortion was considered a risk factor for severe maternal morbidity in the present study, even with borderline statistical significance, as abortion represents a serious public health matter in our country and this information is generally underreported.

The risk of severe maternal morbidity is almost two times higher among women who had less than six prenatal consultations, as recommended by the Ministry of Health. Other studies in Brazil and Argentina, involving severe maternal morbidity cases also found a high prevalence of insufficient number of prenatal consultations [21,26,27]. Although 92% of the cases and 95% of the controls informed having had at least one prenatal consultation, it is still of concern that around 6% of participants, living in a State Brazilian Capital, where access to health services is greater – did not seek or receive prenatal assistance. Even though the prenatal care in Brazil have largely improved in the past decades, the challenge of providing an adequate quantity and quality of prenatal assistance to all pregnant women persists [22,28].

The most important message of this study, for both clinicians and policymakers, is that an adequate prenatal assistance may contribute to prevent severe maternal morbidity among poor woman, even in the presence of other poor social conditions.

However, the present study has some limitations that must be considered. We have not collected data on intra partum care problems because we think that such problems are too closed to the case definition used in this study. Regarding the data gathered, there may be some divergence between the information obtained from medical charts and those present in prenatal cards, considering that the quality and completeness of these sources can vary. But, if such problem occurred it is unlikely to have been differential, thus biasing the odds ratios found towards the null. As to the information collected in the interviews, we cannot rule out that the women who had a *near miss* experience answered differently from those who did not. Nevertheless, as the cases were interviewed in the same period of the puerperal cycle and close to being discharged, when they were well recovered, we believe that such influence was minimized. The sample used did not have the statistical power to identify as significant risk factors that were infrequent among the women included in the study.

## Conclusions

The results of this study reinforce the importance of providing prenatal care in adequate number and quality in order to identify high risk women and prevent severe morbid events.

## Competing interests

The authors declare that they have no competing interests.

## Authors' contributions

APPM participated in the design of the study, collection of data, analysis, interpretation of data and wrote the first manuscript draft. SMB participated in the design of the study, statistical analysis, interpretation of data, coordination, helped to draft the manuscript and reviewed of manuscript. VMAP participated in the design of the study, statistical analysis, interpretation of data, coordination, helped to draft the manuscript and reviewed of manuscript. PSG, JEC and MXV participated in data collection and reviewing of the manuscript. All authors read and approved the final manuscript.

## Authors' information

APPM. MD, Doctor of Public Health, Universidade Federal do Maranhão (UFMA), Sao luis, Maranhao, Brazil

SMB. MD, PhD. Full professor, Faculty of Medicine, Universidade Federal de Minas Gerais (UFMG), Belo Horizonte, Minas Gerais, Brazil.

VMAP. MD, PhD. Associate professor, Faculty of Medicine, Universidade Federal de Minas Gerais (UFMG), Belo Horizonte, Minas Gerais, Brazil.

PSG. MD, MSc. Lecturer of Obstetrics, Ceuma Universidade, São Luis, Maranhão, Brazil.

JEC. MD. Gynecologist, University Hospital, Universidade Federal do Maranhão (UFMA), Sao luis, Maranhao, Brazil

MXV. MD. Gynecologist, University Hospital, Universidade Federal do Maranhão (UFMA), Sao luis, Maranhão, Brazil

SMB and VMAP are research fellows of the National Research Council (CNPQ, grant no. 300908/95 and 300159/99-4).
